# Sex-Biased Gene Expression and Isoform Profile of Brine Shrimp *Artemia franciscana* by Transcriptome Analysis

**DOI:** 10.3390/ani11092630

**Published:** 2021-09-07

**Authors:** Euna Jo, Seung-Jae Lee, Eunkyung Choi, Jinmu Kim, Jun-Hyuck Lee, Hyun Park

**Affiliations:** 1 Division of Biotechnology, College of Life Sciences and Biotechnology, Korea University, Seoul 02841, Korea; eunajo@kopri.re.kr (E.J.); skullcap@korea.ac.kr (S.-J.L.); amy_choi@korea.ac.kr (E.C.); rlawlsan04@korea.ac.kr (J.K.); 2 Division of Life Sciences, Korea Polar Research Institute (KOPRI), Incheon 21990, Korea; 3 Research Unit of Cryogenic Novel Material, Korea Polar Research Institute (KOPRI), Incheon 21990, Korea; 4 Department of Polar Sciences, University of Science and Technology, Incheon 21990, Korea

**Keywords:** *Artemia franciscana*, transcriptome, isoforms, sex-biased gene expression, sex determination

## Abstract

**Simple Summary:**

The brine shrimp *Artemia* is a promising model organism for ZW sex determination system, but the genes related to sex determination and differentiation of *Artemia* have not yet been examined in detail. In this study, the first isoform-level transcriptome sequencing was performed on female and male *Artemia franciscana*. By using PacBio Iso-Seq and Illumina RNA-Seq technologies, we found 39 candidate sex determination genes that showed sex-biased gene expression. The male-biased expressed genes included *DMRT1* and *Sad* genes, which had three and seven isoforms, respectively. Among these, the *Sad* gene is an ecdysteroid biosynthetic pathway gene associated with arthropod molting and metamorphosis. We propose the importance and the necessity of further research on genes involved in ecdysteroid biosynthesis. These results will contribute to understand sex determination and differentiation of *Artemia* and other crustaceans having ZW systems.

**Abstract:**

The brine shrimp *Artemia* has a ZW sex determination system with ZW chromosomes in females and ZZ chromosomes in males. *Artemia* has been considered a promising model organism for ZW sex-determining systems, but the genes involved in sex determination and differentiation of *Artemia* have not yet been identified. Here, we conducted transcriptome sequencing of female and male *A. franciscana* using PacBio Iso-Seq and Illumina RNA-Seq techniques to identify candidate sex determination genes. Among the 42,566 transcripts obtained from Iso-Seq, 23,514 were analyzed. Of these, 2065 (8.8%) were female specific, 2513 (10.7%) were male specific, and 18,936 (80.5%) were co-expressed in females and males. Based on GO enrichment analysis and expression values, we found 10 female-biased and 29 male-biased expressed genes, including *DMRT1* and *Sad* genes showing male-biased expression. Our results showed that *DMRT1* has three isoforms with five exons, while *Sad* has seven isoforms with 2–11 exons. The *Sad* gene is involved in ecdysteroid signaling related to molting and metamorphosis in arthropods. Further studies on ecdysteroid biosynthetic genes are needed to improve our understanding of *Artemia* sex determination. This study will provide a valuable resource for sex determination and differentiation studies on *Artemia* and other crustaceans with ZW systems.

## 1. Introduction

Most animals develop into two sexes, male or female, which are generally determined by a pair of sex chromosomes. In contrast to the male-heterogametic (XY) sex determination system found in most mammals, some organisms, including birds, snakes, fish, insects, and crustaceans, have a female-heterogametic (ZW) sex determination system [1]. Sex determination is an interesting theme, especially for researchers studying evolution and development. Accordingly, many studies have been conducted on sex-determining genes in several organisms, such as mammals [2,3,4], birds [5,6,7], insects [8], and crustaceans [9,10,11,12,13,14,15]. Primary sex determination depends on the sex chromosomes and is responsible for gonadal determination [16]. Well-known primary sex-determining genes are the sex-determining region of the Y chromosome (*SRY*) gene in humans [17] and the Z-linked doublesex and mab-3-related transcription factor 1 (*DMRT1*) gene in chickens [5]. Regarding secondary sex determination, sexual phenotypes are regulated by sex hormones or sex steroids secreted from gonads in vertebrates [16,18,19]. Ecdysteroids, the molting hormones in invertebrates, have been proposed to play roles as sex hormones in secondary sex determination [20,21]. They are suggested to be involved in sexual dimorphism and have much wider functions in insects [20,22,23] and crustaceans [24,25,26,27,28].

The brine shrimp *Artemia* is a member of the branchiopod crustaceans and is distributed worldwide in various saline habitats [29]. In the genus, seven species undergo bisexual reproduction, and numerous parthenogenetic populations, also known as *A. parthenogenetica*, can reproduce asexually [30,31,32]. Female and male *Artemia* are easily distinguished by their phenotypes. Females are usually 1–2 mm larger than males and have an ovisac. Males have a paired penis and markedly enlarged second antennae [33,34]. The genus *Artemia* has a ZW sex determination system, which means ZW chromosomes in females and ZZ chromosomes in males [35,36]. Since Bowen [37] first proposed the heterogametic sex chromosomes in *Artemia* females through sex-linked mutation experiments, the ZW system of sex determination has been supported and confirmed by amplified fragment length polymorphism (AFLP)-based linkage maps [36] and Z-linked gene expression analysis [35]. The sex-determining genes in *Artemia* have not yet been identified; however, some genes related to sexual differentiation have been identified. *DMRT*, or doublesex (*Dsx*), which is widely expressed in many vertebrates, insects, and crustaceans [12,15,38,39,40,41], has been suggested to be involved in sexual differentiation in *Artemia* [42]. The CCCH-type zinc finger gene Masculinizer (*Masc*), which was proposed to control the sex-specific expression of the *Dsx* gene of the silkworm *Bombyx mori* [43], has also been identified in *Artemia* [33]. Nevertheless, a recent study revealed that these genes are located in the autosomal region and are not primary sex determination genes [35]. It is implied that there may be different master sex determination genes unknown in other animals, so further studies on the sex determination genes of *Artemia* are required.

Recently, many studies on sex determination/differentiation genes through transcript expression profiles have made widespread use of long-read sequencing platforms. In particular, PacBio Iso-Seq can generate full-length transcripts without assembly, allowing splicing variants of complex isoforms to be revealed [44,45,46]. Individual genes generate various mRNA isoforms by alternative splicing, which can produce multiple functional proteins [47]. Therefore, it is necessary to conduct a transcriptome study at the isoform level. To date, sex-related transcriptome research of *A. franciscana* has been conducted using the 454 GS FLX titanium platform [48] and Illumina HiSeq system [35]. However, no transcriptome studies have been conducted on full-length transcripts at the isoform level in *Artemia*. In the present study, we sequenced *A. franciscana*, a bisexual *Artemia* species, using PacBio Iso-Seq and Illumina RNA-Seq technologies to identify genes with sex-biased expression, thereby attempting to identify candidate sex determination genes. The genus *Artemia* is considered to be a model organism for studying ZW sex-determining systems due to its short life cycle; ease of raising; and the appearance of two reproductive strategies, sexual and asexual [33,35,36]. Moreover, *Artemia* has utility as an organism for toxicological testing and as a food source for aquaculture industries [49,50,51,52]. This study provides a basis for the sex-biased gene expression and isoform-level transcriptome study of *Artemia* and helps to understand sex determination and differentiation in crustacean species using the ZW sex determination system.

## 2. Materials and Methods

### 2.1. Sample Preparation and RNA Extraction

**Figure 1:**
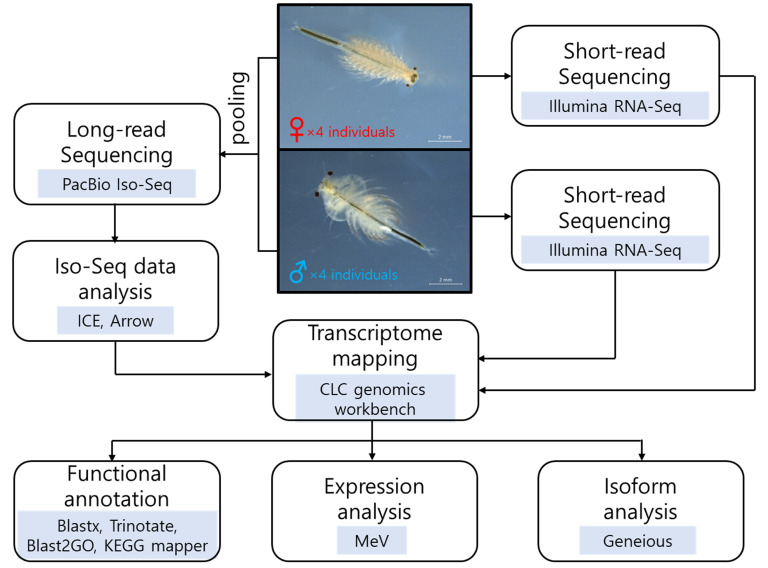
Workflow of the bioinformatic steps. The photographs of female and male *Artemia franciscana* (scale bar = 2 mm), and the flowchart showing analysis tools used in this study.

*Artemia franciscana* were hatched from commercial cysts (INVE Technologies NV, Dendermonde, Belgium) and cultured in 30 g/L saline water at 25 °C with aeration. The live green alga *Tetraselmis* sp. was used as a food source. Adult females and males of *A. franciscana* were collected separately, mainly based on the size of the second antennae (Figure 1). Before RNA extraction, they were not fed for 3 days to reduce the influence of their food. After washing three times with distilled water, four pooled females and males were homogenized in TRIzol Reagent (Ambion, Austin, TX, USA) followed by total RNA extraction using the Direct-zol RNA MicroPrep Kit (Zymo Research, Irvine, CA, USA). RNA quantity and quality were measured using a Qubit fluorometer (Invitrogen, Life Technologies, Carlsbad, CA, USA) and Fragment Analyzer (Agilent Technologies, Santa Clara, CA, USA), respectively.

### 2.2. Iso-Seq Sequencing for Full-Length Transcripts Generation

Before constructing the PacBio Iso-Seq library, RNAs from different sexes of *A. franciscana* were mixed in equal amounts. Complementary DNA (cDNA) was synthesized using the SMARTer PCR cDNA Synthesis Kit (Clontech, CA, USA). The Iso-Seq SMRTbell library was prepared using a DNA template prep kit 1.0 (Pacific Biosciences, Menlo Park, CA, USA) according to the manufacturer’s protocol. The library was sequenced using two SMRT cells and Sequel Sequencing Chemistry 3.0 (Pacific Biosciences, Menlo Park, CA, USA) on the Sequel platform with 20 h movie times. Raw data were processed using SMRT Link v8.0.0 with the Iso-Seq3 application (Pacific Biosciences, Menlo Park, CA, USA). Circular consensus sequences (CCSs) were built from subreads and classified into full-length non-chimeric (FLNC) reads or non-full length (NFL) reads by identifying polyA/T tails and 5′ and 3′ primers. The FLNC reads were clustered using iterative clustering for error correction (ICE) algorithm to produce consensus isoforms and then polished with NFL reads based on an Arrow accuracy of 99%.

### 2.3. RNA-Seq Sequencing and Mapping to Iso-Seq Data

Illumina RNA-Seq libraries were prepared individually for female and male RNAs, which were the same as those used for the Iso-Seq library construction. Following the standard protocol, paired-end libraries were constructed using a Truseq Stranded mRNA Prep kit (Illumina, San Diego, CA, USA) and sequenced on the Illumina NovaSeq 6000 platform. The quality of the raw data was assessed using the FastQC application [53]. Using CLC Genomics Workbench v12.0.3 (QIAGEN, Aarhus, Denmark), the female and male RNA-Seq data were severally mapped to the 42,566 transcripts generated from PacBio Iso-Seq. The expression levels were calculated as transcripts per million (TPM) values for each transcript. A TPM cutoff value of 1 was used to exclude transcripts with no or extremely low expression. Transcripts with a TPM ≥ 1 exclusively in one sex were considered sex-specific transcripts.

### 2.4. Functional Annotation and Sex-Biased Expression Analysis

The full-length transcripts generated by Iso-Seq were annotated using BLASTX [54] by aligning to the NCBI non-redundant (nr) protein database and Trinotate v2.3.0 pipeline [55]. Gene ontology (GO) term mapping and annotation were performed in terms of the biological process (BP), molecular function (MF), and cellular component (CC) using Blast2GO v5.0 [56,57]. To avoid contaminant sequences from the culture environment, the transcripts belonging to plants, algae, bacteria, and viruses were filtered out based on the BLAST best-hit species. For the remaining female and male-specific transcripts, GO enrichment analysis with a two-tailed Fisher’s exact test (*p* < 0.05) was conducted using OmicsBox v1.3.11 [56,58]. The heatmaps were displayed based on TPM values for transcripts assigned to the top two BP GO terms, which were enriched specifically in females or males, using MultiExperiment Viewer (MeV) v4.9.0 [59]. Kyoto Encyclopedia of Genes and Genomes (KEGG) pathway mapping was performed using the KEGG Automatic Annotation Server (KAAS) [60] and KEGG Mapper [61]. The term ‘sex-biased genes’ refers to genes that are expressed only in one sex (sex-specific) or are more highly expressed in one sex (sex-enriched) [62].

## 3. Results

### 3.1. Transcriptome Sequencing Results of Female and Male A. franciscana

To identify transcripts differentially expressed in adult females and males of *A. franciscana,* Iso-Seq and RNA-Seq were performed, and the data were analyzed following the bioinformatic steps shown in Figure 1. We used four adult individuals (i.e., four females or four males) for RNA extraction to minimize sample variation. The Iso-Seq library was prepared by pooling both RNAs, whereas RNA-Seq libraries were constructed independently for each RNA. Iso-Seq sequencing generated 678,253 subreads with a total read length of 2,128,734,891 bp and a mean length of 3138 bp (Table S1). A total of 42,566 high-quality isoforms were obtained after clustering and polishing the Iso-Seq data (Table S1). RNA-Seq sequencing produced 113 million reads with a total yield of ~11.4 Gb for females and 114 million reads with a total yield of ~11.5 Gb for males (Table S1).

We decided the sex-specific transcriptome sets by choosing transcripts with TPM values of one or higher in only one sex and filtering out the putative contaminant sequences. Consequently, a total of 23,514 out of 42,566 transcripts remained, among which 2065 (8.8%) were female specific, 2513 (10.7%) were male specific, and 18,936 (80.5%) were common in females and males (Figure S1, Table S2). The number of male-specific transcripts was higher than that of female-specific transcripts in *A. franciscana*.

### 3.2. Functional Annotation and GO Enrichment Analysis

**Figure 2:**
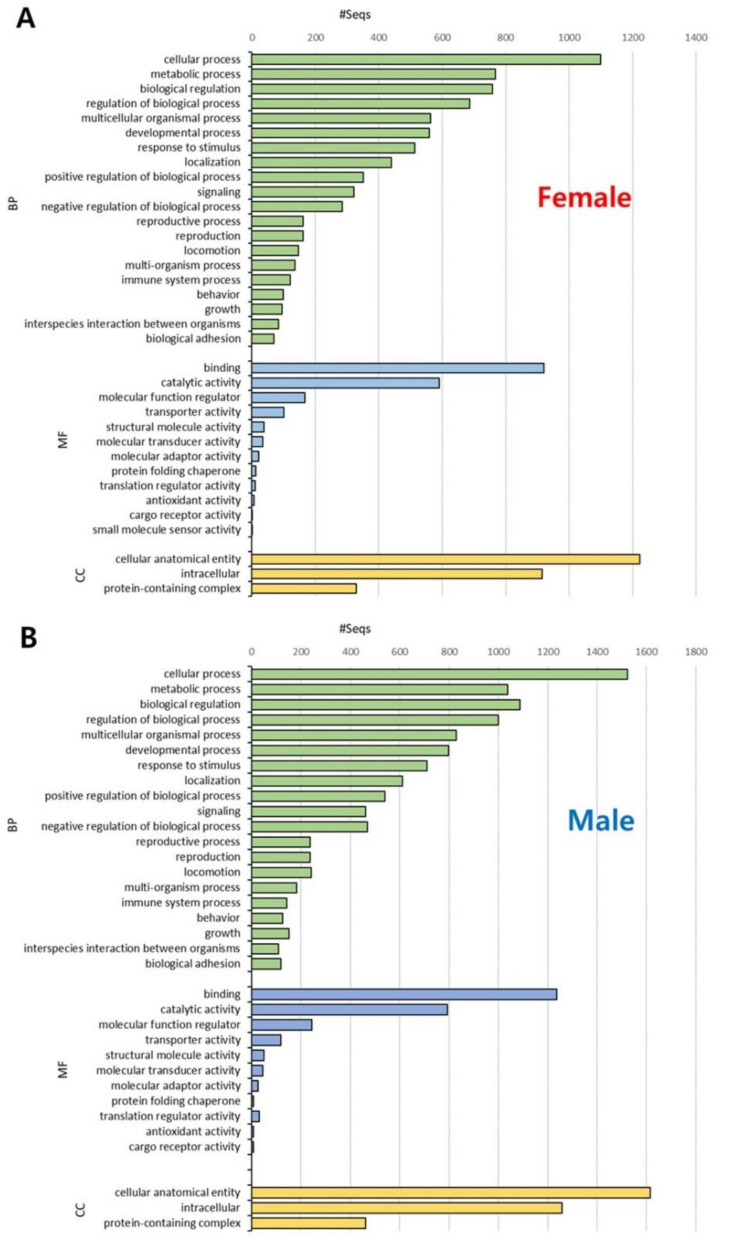
Gene Ontology (GO) distribution of sex-specific transcripts in *Artemia franciscana* transcriptome. Functional annotation was conducted for female-specific transcripts (**A**) and male-specific transcripts (**B**). GO terms were divided into three major categories: biological process (BP, green), molecular function (MF, blue), and cellular component (CC, yellow). The *x*-axis indicates the number of transcripts, and the *y*-axis indicates level 2 GO terms.

To compare functional information between sex-specific transcripts in *A. franciscana*, GO analysis was conducted for three categories (i.e., BP, MF, and CC). First, GO-term distributions were analyzed by level 2 for 2065 female- and 2513 male-specific transcripts, respectively. In the BP category, the majority of transcripts were related to ‘cellular process’ GO terms in both females and males, followed by ‘metabolic process’ in females and ‘biological regulation’ in males (Figure 2). Additionally, the transcripts were the most distributed in the binding (MF) and cellular anatomical entity (CC) GO terms in both sexes (Figure 2). The overall GO types and proportions showed similar patterns between females and males, although there were slight differences in the BP and MF categories.

**Figure 3:**
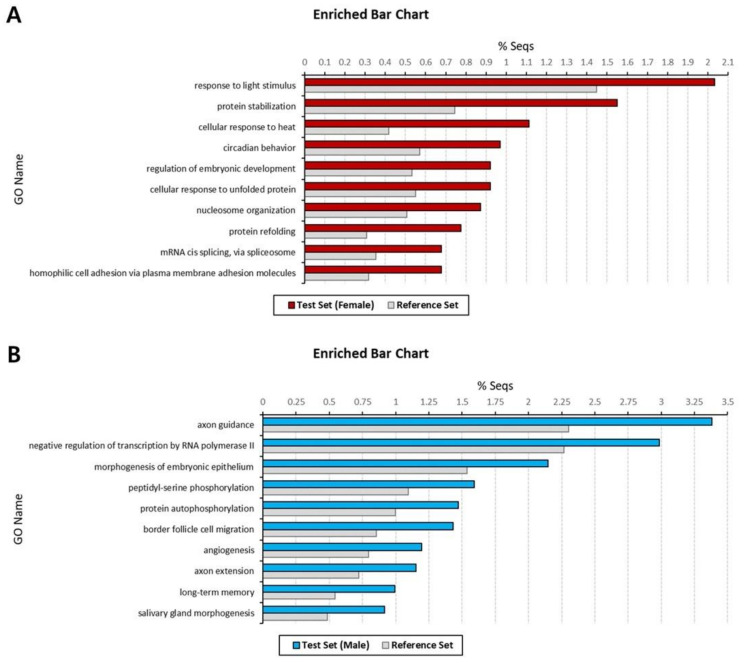
Top 10 enriched Gene Ontology (GO) terms of sex-specific transcripts in *Artemia franciscana* transcriptome. Enrichment analysis was performed for female-specific transcripts (**A**) and male-specific transcripts (**B**). Biological process GO terms that exhibit statistically significant differences are shown in the figure (Fisher’s exact test, *p* < 0.05). The X-axis represents the relative frequencies, and the Y-axis represents significant enriched GO terms.

Next, we performed GO enrichment analysis for the female- and male-specific transcripts, with *p*-value < 0.05, to investigate which GO terms were sex specific and over-represented. After filtering to reduce to the most specific GO terms, the numbers of significantly enriched GO terms were 84 BP, 36 MF, and 19 CC in females and 209 BP, 60 MF, and 43 CC in males (Tables S3 and S4). Among these, the top 10 BP GO terms, which contain the largest number of transcripts, are presented in Figure 3. In the female set, the most abundant GO terms were ‘response to light stimulus’ (2.04%), ‘protein stabilization’ (1.55), and ‘cellular response to heat’ (1.11%) (Figure 3A). For the male set, the most enriched GO terms included ‘axon guidance’ (3.38%), ‘negative regulation of transcription by RNA polymerase II’ (2.98%), and ‘morphogenesis of embryonic epithelium’ (2.15%) (Figure 3B).

### 3.3. Sex-Biased Gene Expression Profile and Candidate Sex Determination Genes

**Figure 4:**
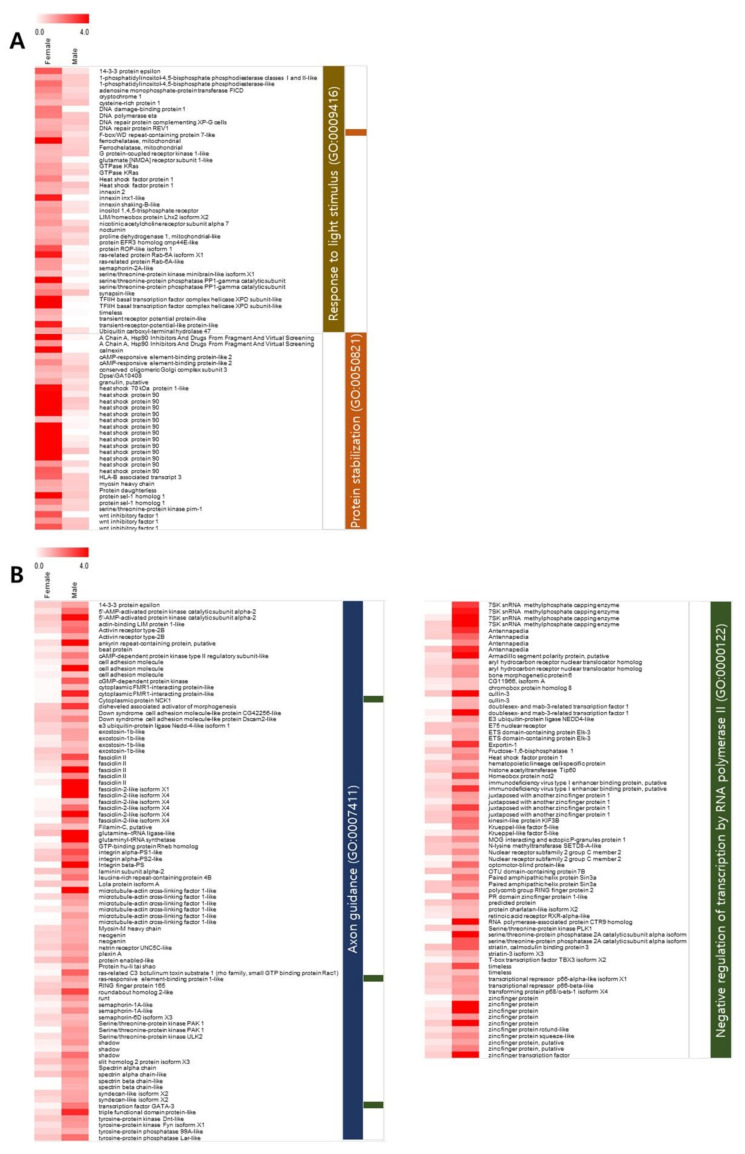
Heatmap of sex-biased expressed genes involved in top two enriched Gene Ontology (GO) terms. Heatmaps were generated for female-specific transcripts (**A**) and male-specific transcripts (**B**). Each line represents a transcript annotated by BLAST, and the color scale indicates relative expression level by transcripts per million (TPM) values.

In this study, we identified genes with sex-biased expression to predict candidate sex determination genes in *A. franciscana*. First, sex-biased transcripts assigned to the enriched GO terms were examined, especially for the major GO terms belonging to the BP category. The numbers of transcripts selected for subsequent analysis were as follows: 42 transcripts in ‘response to light stimulus’ (GO:0009416) and 32 transcripts in ‘protein stabilization’ (GO:0050821) in females and 85 transcripts in ‘axon guidance’ (GO:0007411) and 75 transcripts in ‘negative regulation of transcription by RNA polymerase II’ (GO:0000122) in males. Heatmaps for these transcripts, generated based on the TPM values and BLAST annotation, show differential expression between females and males (Figure 4).

We used two criteria to identify the sex-differentially expressed genes among the full-length transcripts: (1) the transcripts had a TPM value of at least two, which were considered ‘highly’ expressed specifically in one sex, and (2) the corresponding gene names contained only female- or male-specific isoforms based on the BLAST description. The number of identified genes with sex-differential expression was 10 in females and 29 in males (Table 1). Of the listed genes, *DMRT1* and Shadow (*Sad*) showing male-biased expression were focused on in further isoform analyses.

### 3.4. Isoforms of DMRT1 and Sad Genes in A. franciscana

The mRNA isoforms of *DMRT1* and *Sad* genes were mapped to the genome assembly (unpublished data) using Geneious v9.1.2 (Biomatters Ltd., Auckland, New Zealand) and compared. Our results showed that three male-biased *DMRT1* isoforms and one *Dsx* isoform are expressed in both sexes (Table S5). Isoforms of the *DMRT1* gene were 3191–3960 bp in length with five exons, of which only the lengths of the fifth exon were different (Figure S2, Table S5). The isoform of the *Dsx* gene was 1497 bp in length with six exons (Figure S2, Table S5).

Regarding *Sad* genes, seven male-biased isoforms were identified, out of which five isoforms showed only male-specific expression (Figure S2, Table S5). Isoforms of the *Sad* gene were 1865–4319 bp in length with 2 to 11 exons and showed various alternative splicing patterns (Figure S2). Ecdysteroid signaling, including the *Sad* gene, is associated with sexual dimorphism in addition to molting and metamorphosis in arthropods. Thus, we focused on the presence and expression of the genes that mediate ecdysteroid biosynthesis, namely Halloween genes, in *A. franciscana*.

### 3.5. KEGG Pathway for Ecdysteroid Biosynthesis

**Figure 5:**
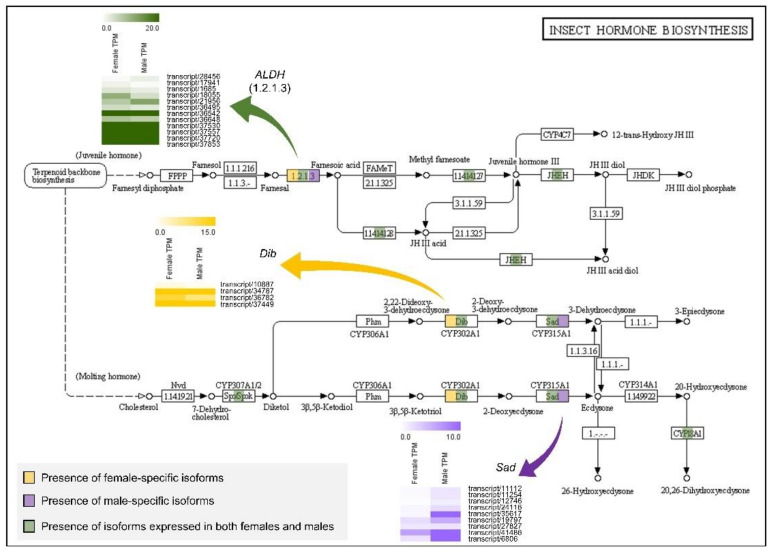
Expression differences in female and male *Artemia franciscana* transcriptome for the insect hormone biosynthesis KEGG pathway. Colored boxes in the Kyoto Encyclopedia of Genes and Genomes (KEGG) pathway indicate the presence of female-specific isoforms (yellow), male-specific isoforms (purple), and isoforms expressed in both sexes (green). Heatmaps represent the expression patterns of *ALDH* (1.2.1.3, green), *Dib* (yellow), and *Sad* (purple) genes. The color scales indicate relative expression levels by transcripts per million (TPM) values. ALDH, aldehyde dehydrogenase; Dib, disembodied; Sad, shadow.

KEGG pathway analysis for insect hormone biosynthesis showed that *A. franciscana* harbored the spook (*Spo*), spookier (*Spok*), disembodied (*Dib*), and *Sad* genes among typical Halloween genes, whereas the phantom (*Phm*) gene did not appear (Figure 5). The *Dib* gene expressed a female-specific isoform, and the *Sad* gene expressed five male-specific isoforms (Figure 5). Additionally, the aldehyde dehydrogenase (*ALDH*) gene in the juvenile hormone (JH) biosynthetic pathway expressed one female- and one male-specific isoform, respectively (Figure 5).

## 4. Discussion

In the present study, we conducted transcriptome sequencing at the isoform level and compared sex-biased gene expression of brine shrimp *A. franciscana* by combining PacBio IsoSeq and Illumina RNA-Seq. As a result of *A. franciscana* transcriptome sequencing, the number of male-specific transcripts (2513) was higher than that of female-specific transcripts (2065) (Figure S1). Sex-biased genes with male-biased expression tend to evolve faster, resulting in more functional divergence than those with female-biased expression [62,63]. This tendency, thought to contribute to sexual selection, is observed in various organisms with the XY system, such as *Drosophila* and *Caenorhabditis elegans*, as well as in organisms with the ZW system, such as birds [62,63,64]. In case of crustaceans, transcriptomic analysis of the Norway lobster *Nephrops norvegicus* in multiple tissues showed a higher number of male-specific transcripts compared with females [14]. However, the opposite results appeared in Pacific white shrimp *Litopenaeus vannamei* and water flea *Daphnia* species [65,66,67]. Thus, further discussion on the evolutionary rates of sex-biased genes in crustaceans is indicated.

Based on GO enrichment analysis and TPM expression values, we found 39 candidate sex-determining genes (Table 1). Of the candidate genes, 29 genes showed male-biased expression, representatively *DMRT1* and *Sad* genes. The *DMRT* or *Dsx* gene has been reported to be involved in sex determination and differentiation in diverse species, including *Artemia*, and is well established as dominant in male sexual differentiation [42]. A novel *DMRT* gene in Chinese mitten crab *Eriocheir sinensis* played an essential role in testicular differentiation and was only expressed in testis [41]. *Dsx* homologous genes of freshwater prawn *Macrobrachium rosenbergii* and *D. magna* were suggested to play roles in male gonad development, although some of the transcripts were expressed in both testis and ovaries [12,39,40]. Farazmand et al. [42], using bisexual *A. urmiana* and asexual *A. parthenogenetica*, demonstrated that *DMRT* genes are involved in female ovarian development but not in male testicular differentiation. This was an unusual result, suggesting the need for research on alternative spliced forms of the *DMRT* family. In this study, one *Dsx* and three *DMRT1* isoforms were identified with higher or exclusive expression in males without female-specific isoforms (Figure S2, Table S5), which seems to be related to male differentiation as in other organisms. Therefore, further studies of *DMRT1* isoforms are needed to identify whether there is a difference in expression depending on the various *Artemia* species.

The *Sad* gene is a member of the Halloween gene family, which encodes the cytochrome P450 enzymes that mediate the ecdysteroidogenic pathway (steroid hormone biosynthesis) [68]. Ecdysteroid signaling, including the *Sad* gene, is associated with sexual dimorphism in addition to molting and metamorphosis in insects. For example, ecdysteroids contribute to extreme sexual dimorphisms in mealybugs, whose mRNA expression levels from the second nymphal instar to adult stages were higher in males than in females [69]. In beetles, ecdysteroids have been reported to control horn length dimorphisms in male heads [70], and Halloween genes, including Shade (*Shd*) and *Phm*, have been suggested as candidate target genes that are downregulated by the *Dsx* gene [71]. These Halloween genes are found not only in insects but also in crustaceans [72,73]. In *D. pulex*, which belongs to *Branchiopoda* along with *Artemia*, orthologs of typical Halloween genes (i.e., *Spo/Spok*, *Phm*, *Dib*, *Sad*, and *Shd*) were presented and conserved [72]. In our results, most of the Halloween genes were retained in *A. franciscana* transcriptome except for the *Phm* gene (Figure 5). Among them, *Dib* and *Sad* genes had one female- and five male-specifically expressed isoforms, respectively (Figure S2, Table S5). Taken together, there may be differences in the expression pattern of Halloween genes among arthropods. The functions and roles of Halloween genes in *Artemia* have not yet been studied extensively; hence, additional validation studies on ecdysteroid biosynthetic pathway genes should be conducted.

Although many studies on sex determination/differentiation on crustaceans have been conducted, compared to active studies on *Decapoda* (e.g., crabs, lobsters, and shrimps), research on *Branchiopoda* is still limited to *Daphnia*. Species that are easy to grow and have availability of genome sequences and embryonic developmental data are useful as new model organisms for crustaceans [74]. As mentioned in the introduction, *Artemia* has several advantages as a model organism for studying sex determination systems. If chromosomal-level genome sequencing of *Artemia* proceeds in the future, it would be possible to reveal the primary sex determination gene located on the sex chromosome. Furthermore, subsequent transcriptome research according to the embryonic and larval developmental stages would help understand the sex determination system and sexual differentiation of *Artemia* more clearly.

## 5. Conclusions

Here, we aimed to investigate genes with sex-biased expression and to suggest candidate sex determination genes in *A. franciscana* by transcriptomic analysis using Iso-Seq and RNA-Seq data. We identified *DMRT1* and *Sad* as candidate genes with sex-biased expression, which were related to male sex determination/differentiation in many animals. The *Sad* gene regulates ecdysteroid biosynthesis and is involved in secondary sex determination and sexual dimorphism. Further research on ecdysteroidogenic genes will improve our understanding of sex determination in *Artemia*. To the best of our knowledge, this is the first isoform-level transcriptome study of *Artemia*. These results will be a valuable resource for the study of sex-biased gene expression in *Artemia* and other crustaceans with ZW sex determination systems.

## Figures and Tables

**Table 1:** Selected candidate genes with sex-biased expression in the *Artemia franciscana* transcriptome.

F/M	Description	Sex-Biased Isoform	Total Isoform
Female	Calnexin	3	7
	DNA damage-binding protein 1	1	5
	Ferrochelatase, mitochondrial	2	5
	Heat shock protein 90	15	25
	HLA-B associated transcript 3	1	3
	Innexin inx1-like	1	4
	Protein ROP-like isoform 1	1	7
	Ras-related protein Rab-6A isoform X1	2	7
	Synapsin-like	1	2
	TFIIH basal transcription factor complex helicase XPD Subunit-like	2	4
Male	5’-AMP-activated protein kinase catalytic subunit alpha-2	2	5
	7SK snRNA methylphosphate capping enzyme	4	8
	Armadillo segment polarity protein, putative	1	5
	cGMP-dependent protein kinase	1	10
	Cullin 3	2	4
	Cytoplasmic FMR1-interacting protein-like	2	2
	Disheveled associated activator of morphogenesis	1	4
	Doublesex- and mab-3-related transcription factor 1	2	3
	Exportin 1	1	4
	Glutamine–tRNA ligase-like	1	3
	Homeobox protein not2	1	1
	Immunodeficiency virus type I enhancer binding protein, putative	2	5
	Integrin alpha-PS1-like	1	2
	Integrin alpha-PS2-like	1	11
	Integrin beta-PS	2	15
	Kinesin-like protein KIF3B	1	3
	Kruppel-like factor 5-like	1	3
	Leucine-rich repeat-containing protein 4B	1	3
	Microtubule-actin cross-linking factor 1-like	6	29
	Optomotor-blind protein-like	1	2
	Ras-related C3 botulinum toxin substrate 1 (rho family, Small GTP binding protein Rac1)	1	3
	RNA polymerase-associated protein CTR9 homolog	1	3
	Serine/threonine protein phosphatase 2A catalytic subunit alpha isoform	2	8
	Shadow	5	7
	Transcription factor GATA-3	1	4
	Triple functional domain protein-like	7	12
	Tyrosine protein phosphatase Lar-like	1	3
	Zinc finger protein	13	30
	Zinc finger transcription factor	1	5

## Data Availability

The sequencing data produced in this study have been deposited in the NCBI Sequence Read Archive (SRA) under accession numbers SRR14598203–SRR14598205 and BioProject accession number PRJNA449186.

## Supplementary Materials

The following are available online at https://www.mdpi.com/article/10.3390/ani11092630/s1, Figure S1: Venn diagram showing the number of transcripts in female and male *Artemia franciscana*; Figure S2: Illustration of isoforms of *DMRT1*, *Dsx*, and *Sad* genes in *Artemia franciscana*; Table S1: Statistics of Iso-Seq and RNA-Seq sequencing results for *Artemia franciscana*; Table S2: Summary of annotations of *Artemia franciscana* transcriptome; Table S3: Female-specific enriched Gene Ontology (GO) terms; Table S4: Male-specific enriched Gene Ontology (GO) terms; Table S5: Information of isoforms of *DMRT1*, *Dsx*, and *Sad* genes in *Artemia franciscana*.
