# Supplementary material for: Sex-Biased Gene Expression and Isoform Profile of Brine Shrimp Artemia franciscana by Transcriptome Analysis
Source: Animals (Basel). 2021 Sep 7;11(9):2630. doi: 10.3390/ani11092630 (PMC8465105; doi:10.3390/ani11092630)
Supplement: Supplementary file 1 [file animals-11-02630-s001.zip › Figure S1.pdf]

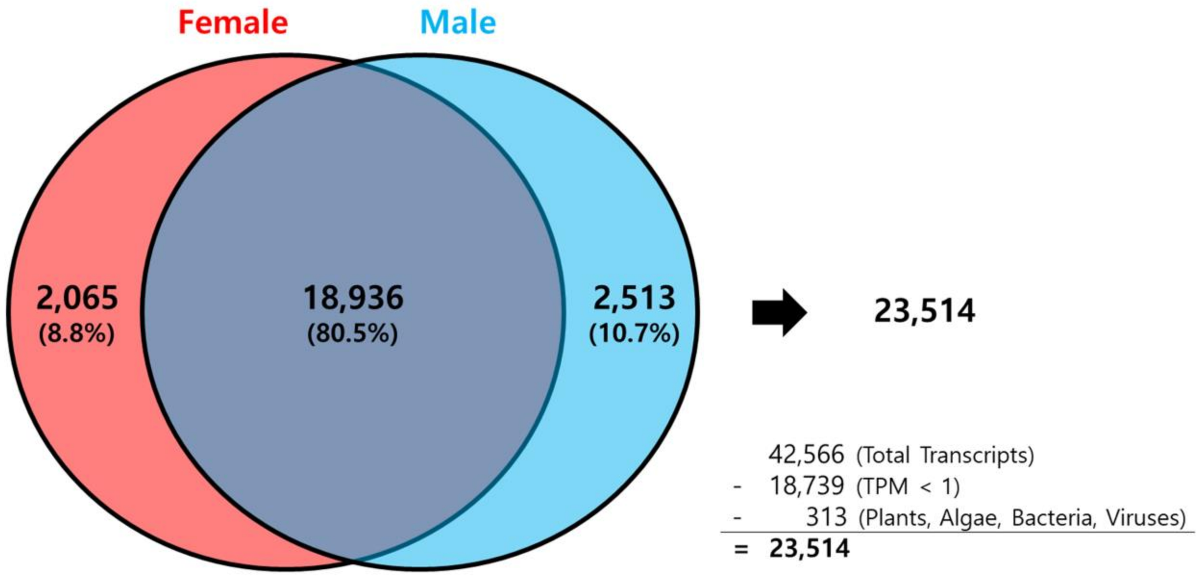

Figure S1. Venn diagram showing the number of transcripts in female and male *Artemia franciscana*. The number of transcripts remaining after filtering out is displayed in the bottom-right. Each color shows female-specific transcripts (red), male-specific transcripts (blue), and transcripts presented in both females and males (navy).
