# Supplementary material for: Sex-Biased Gene Expression and Isoform Profile of Brine Shrimp Artemia franciscana by Transcriptome Analysis
Source: Animals (Basel). 2021 Sep 7;11(9):2630. doi: 10.3390/ani11092630 (PMC8465105; doi:10.3390/ani11092630)
Supplement: Supplementary file 1 [file animals-11-02630-s001.zip › Figure S2.pdf]

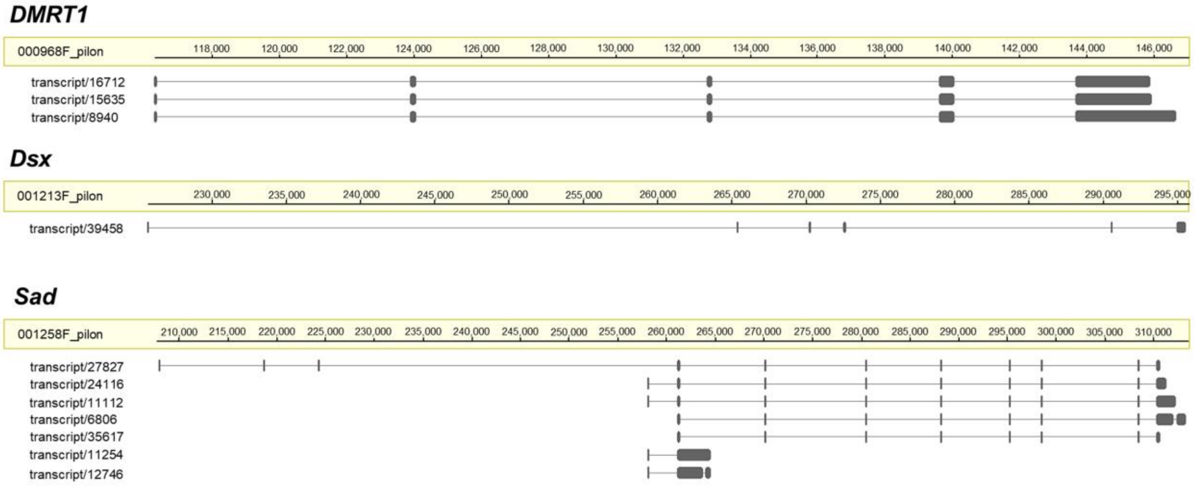

Figure S2. Illustration of isoforms of *DMRT1*, *Dsx,* and *Sad* genes in *Artemia franciscana*. Grey boxes represent exons and the horizontal lines connecting the boxes represent introns.
