# Supplementary figures and images for: Sex-Biased Gene Expression and Isoform Profile of Brine Shrimp Artemia franciscana by Transcriptome Analysis
Source: Animals (Basel). 2021 Sep 7;11(9):2630. doi: 10.3390/ani11092630 (PMC8465105; doi:10.3390/ani11092630)

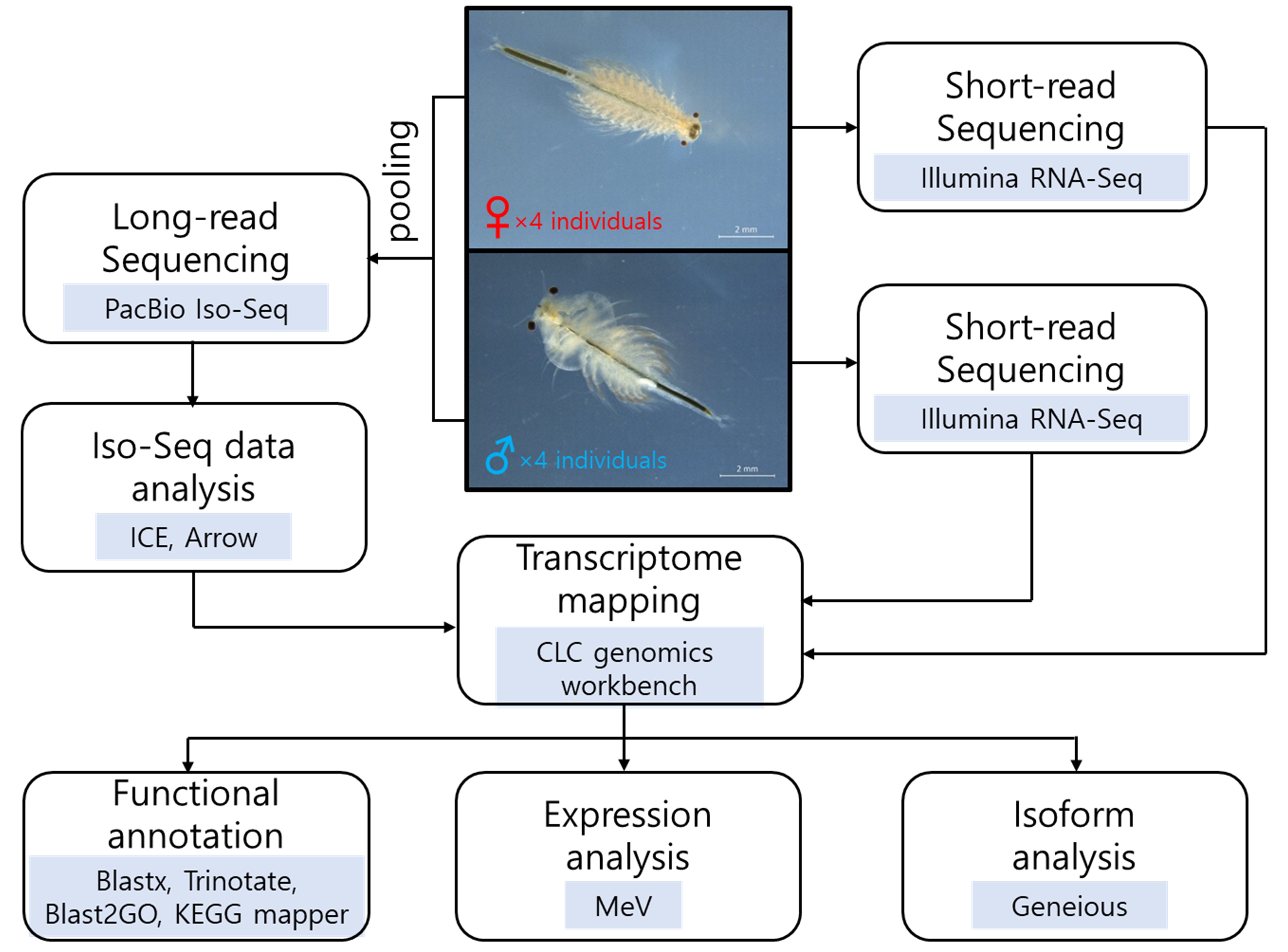

Supplement: Supplementary file 1 [file animals-11-02630-s001.zip › Figure1.jpg]

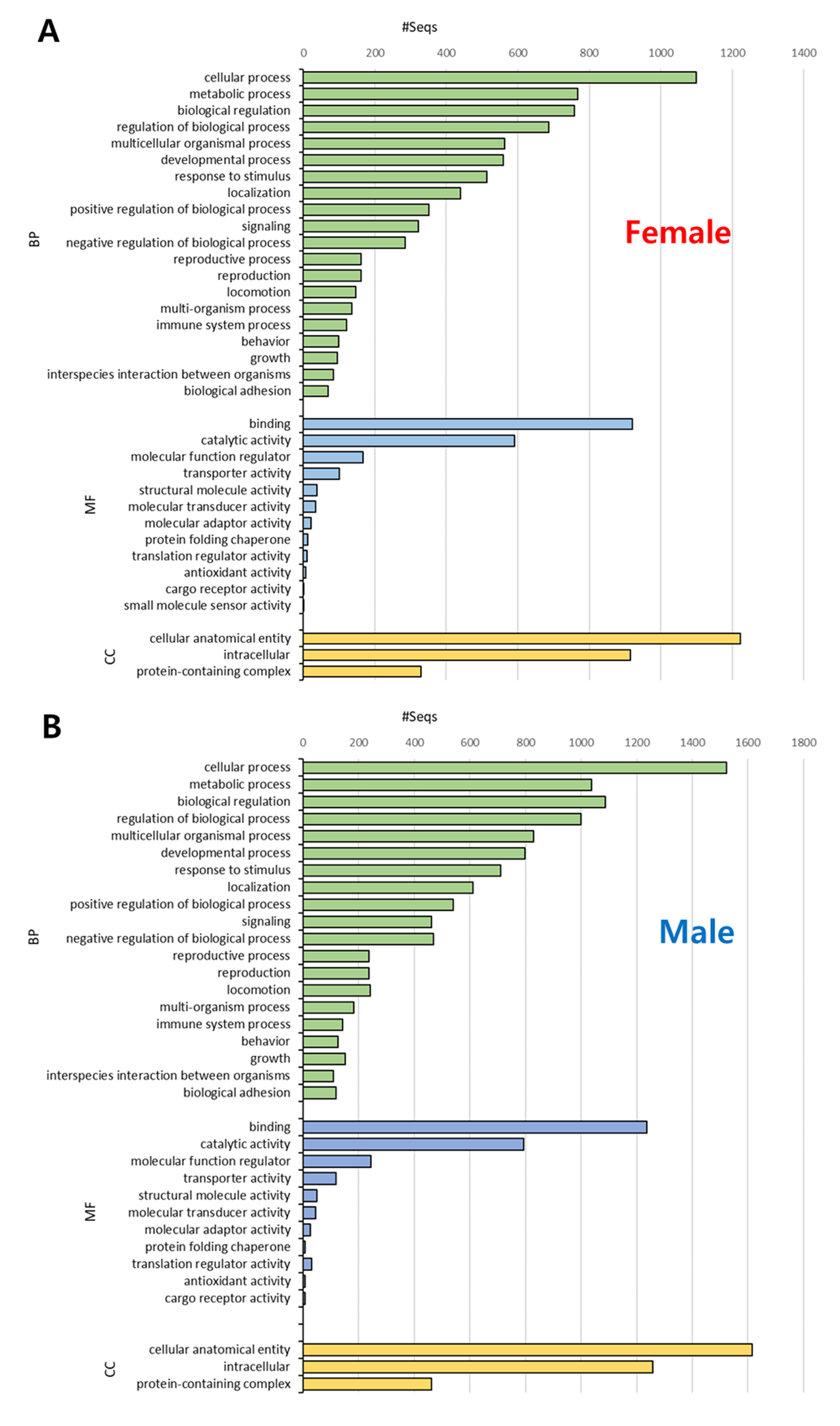

Supplement: Supplementary file 1 [file animals-11-02630-s001.zip › Figure2.jpg]

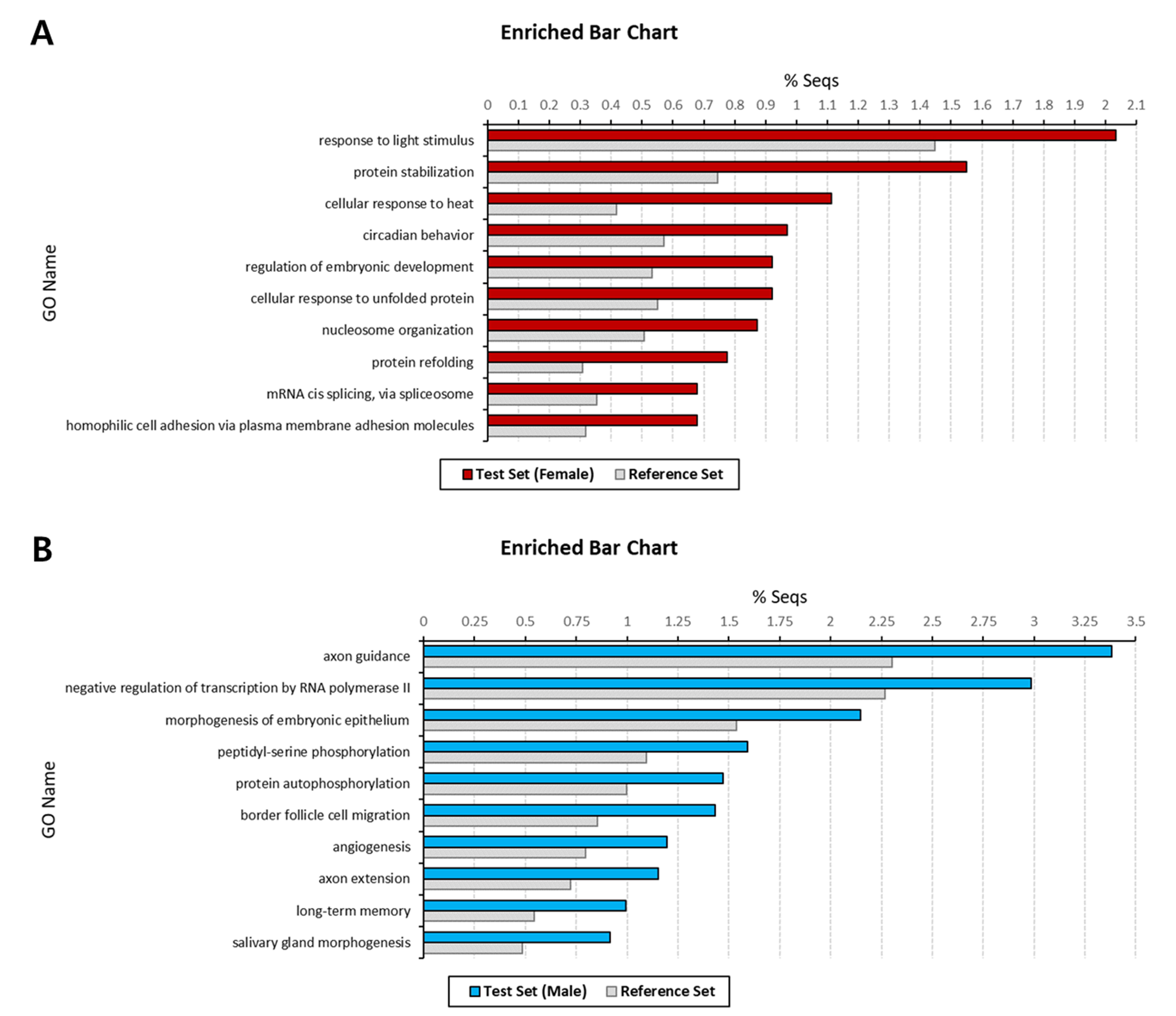

Supplement: Supplementary file 1 [file animals-11-02630-s001.zip › Figure3.jpg]

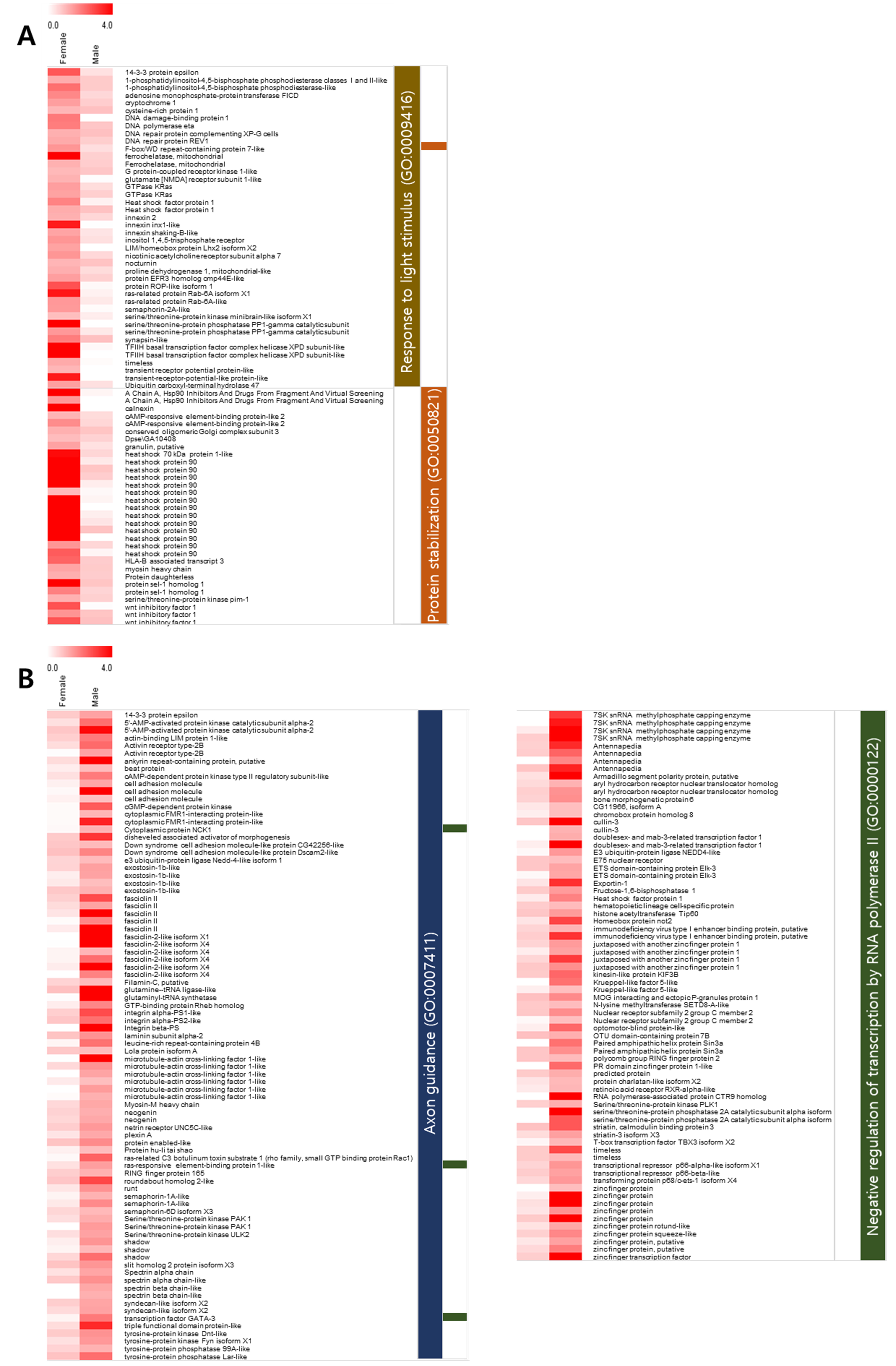

Supplement: Supplementary file 1 [file animals-11-02630-s001.zip › Figure4.jpg]

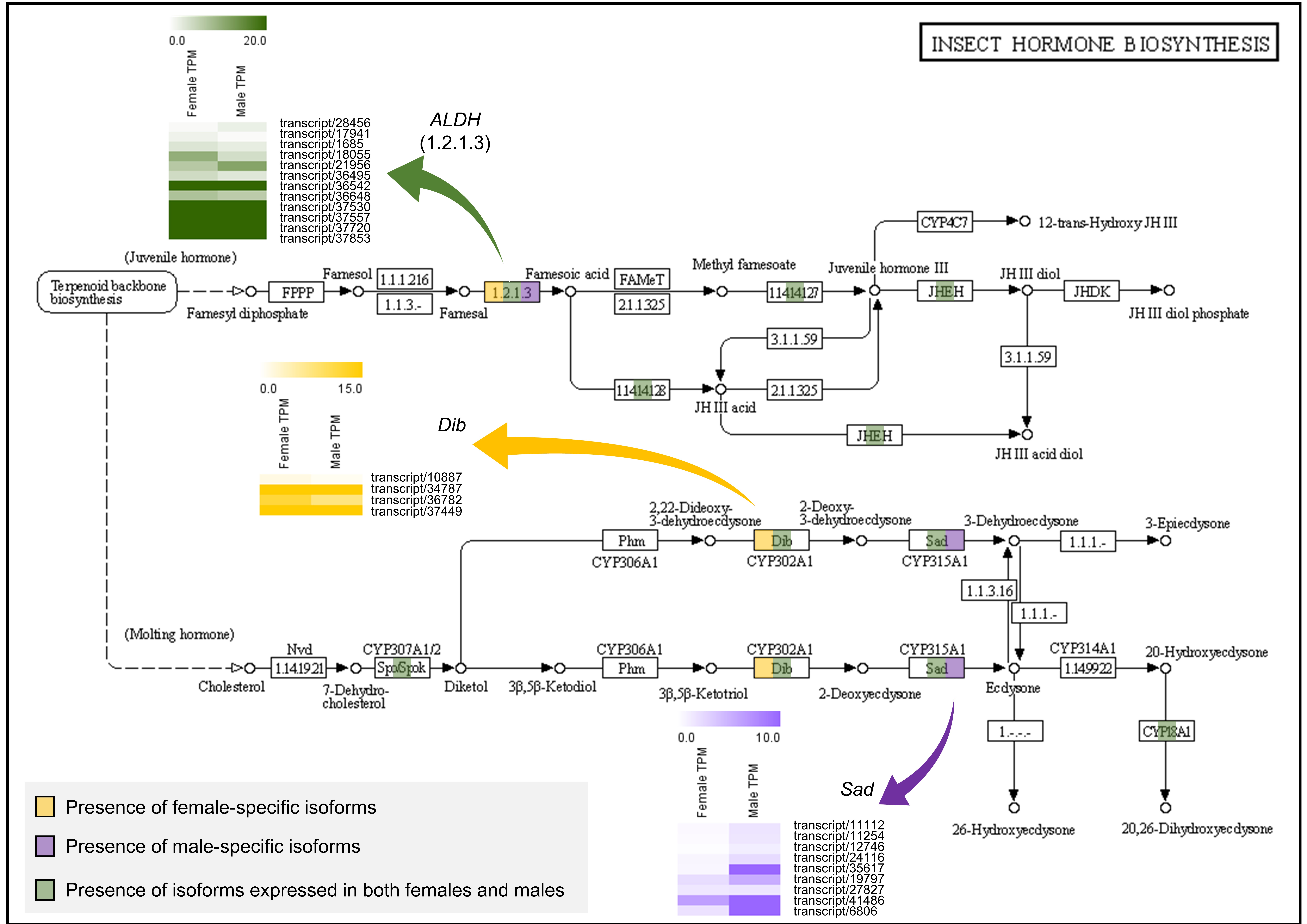

Supplement: Supplementary file 1 [file animals-11-02630-s001.zip › Figure5.jpg]
